# Radiation-Associated Calcific Right Coronary Artery Occlusion Presenting as ST-Elevation Myocardial Infarction: Emphasizing the Need for Early Cardiovascular Screening

**DOI:** 10.7759/cureus.109283

**Published:** 2026-05-20

**Authors:** Khaled Bani Yaseen, Hanin Lataifeh, Wassim Abouzeid, Ahmad W Haddad, Noreen Mirza

**Affiliations:** 1 Internal Medicine, Saint Michael's Medical Center, Newark, USA; 2 Internal Medicine, Unity Health - White County Medical Center, Searcy, USA; 3 Cardiology, Saint Michael's Medical Center, Newark, USA

**Keywords:** breast cancer, coronary artery calcification, heart block, myocardial infarction, radiation therapy

## Abstract

We present the case of a 74-year-old Filipino female patient who presented with ST-elevation myocardial infarction and was found to have severe calcification in the right coronary artery. The patient had a history of breast cancer and had undergone radiation therapy 30 years prior. This case highlights the importance of recognizing and monitoring cardiovascular complications in cancer survivors, particularly those who have received radiation therapy, as they may be at an increased risk of coronary artery calcification and related events such as myocardial infarction.

## Introduction

ST-elevation myocardial infarction (STEMI) is a common and life-threatening manifestation of acute coronary syndrome, typically associated with well-established cardiovascular risk factors such as smoking, hypertension, diabetes mellitus, and dyslipidemia [1]. However, it may also occur in patients with only subtle traditional risk factors [1]. Chest radiation therapy has been increasingly recognized as an important contributor to accelerated coronary artery disease, through inducing arterial wall calcification, with adverse cardiovascular effects that may manifest years to decades after exposure [2,3]. One of the most serious complications is radiation-associated coronary artery calcification (CAC), leading to myocardial infarction (MI) [2].

This case highlights the long-term cardiovascular consequences of chest radiation therapy used in breast cancer treatment and underscores the importance of awareness, surveillance, and further research into optimal screening strategies for cardiovascular disease in cancer survivors [3,4].

## Case presentation

A 74-year-old Filipino female patient presented to the emergency department complaining of chest pain that lasted for 15 minutes and began on the morning of admission. It was substernal and radiated to the left arm. She denied experiencing shortness of breath, palpitations, diaphoresis, nausea, or vomiting episodes.

On physical examination, her blood pressure was 79/50 mmHg, heart rate was 54 beats per minute (bpm), respiratory rate was 20 breaths per minute, oxygen saturation was 97% on room air, and temperature was 37.1°C. The patient was in distress because of her pain. On cardiac auscultation, S1 and S2 were appreciated, with no S3 or S4 heard. Lung auscultation was clear bilaterally with no crackles or wheezes. There was no lower extremity edema.

Medical history, family history, social history, and home medications

Her medical history included hypertension, dyslipidemia, and a history of breast cancer that was treated at age 30 with a radical mastectomy followed by high-dose radiation therapy over two months; she is currently on hormonal therapy. Her family history was significant for essential hypertension involving the patient’s father and mother. Her social history revealed that she did not smoke, drink alcohol, or use recreational drugs. Her home medications included amlodipine 5 mg daily, lisinopril 10 mg daily, metoprolol tartrate 50 mg twice a day, simvastatin 20 mg daily, and anastrozole 1 mg daily.

Diagnostic evaluation

**Figure 1:**
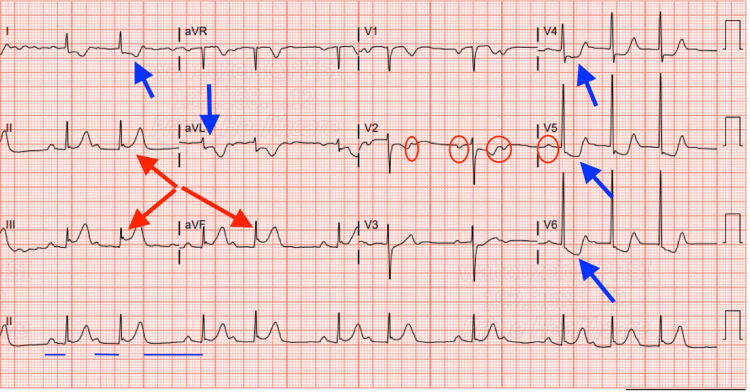
Sinus rhythm with second-degree Mobitz 1 (blue lines) and 2 (red circles), with ST elevations in lead II, III, and avF (red arrows) and reciprocal ST segment depression and T wave inversion involving lead I, avL, V4, V5, and V6 (blue arrows).

The initial electrocardiogram (ECG) showed a sinus rhythm with a heart rate of 70 bpm, alternating between second-degree atrioventricular block types Mobitz 1 and Mobitz 2. It also revealed ST elevations in leads II, III, and aVF, which are most consistent with an inferior wall MI involving the right coronary artery (RCA). Additionally, there was reciprocal ST segment depression and T wave inversion in leads I, aVL, V4, V5, and V6 (Figure 1).

The patient’s complete blood count was unremarkable, with a white blood cell count of 7.20 × 10^3^/µL (reference range: 3.5-10.5 × 10^3^/µL) and a hemoglobin level of 14.1 g/dL (reference range: 12-15.5 g/dL). The initial troponin level was 21.1 ng/L (reference range <14 ng/L), with a B-type natriuretic peptide (BNP) level of 45 pg/mL (normal range <100 pg/mL). Complete metabolic panel, procalcitonin, and C-reactive protein were all within normal range.

Management (including interventional, surgical, and medical, as needed)

**Figure 2:**
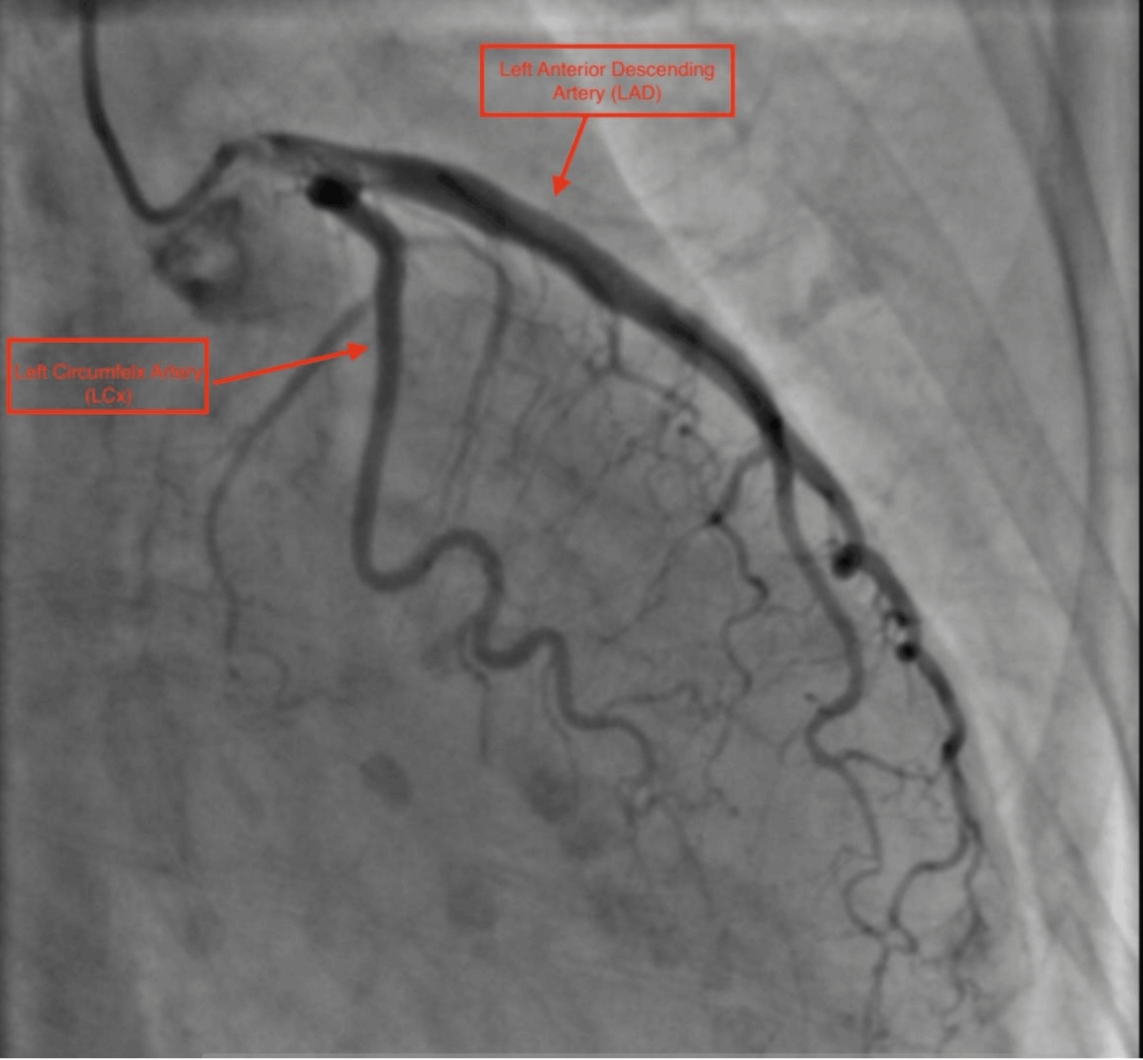
Left circulation: patent LAD artery and LCx with collateral and calcium deposits. LAD, left anterior descending; LCx, left circumflex artery

A loading dose of aspirin, ticagrelor, and intravenous heparin was given, after which the patient was transferred directly after the ECG findings to the cath lab for emergent left heart catheterization and angiography. A catheter was introduced through proper femoral access, and upon evaluation of left coronary circulation, angiography revealed a patent left main coronary artery, left anterior descending (LAD) artery, and left circumflex artery (LCx) with multiple collaterals (Figure 2).

**Figure 3:**
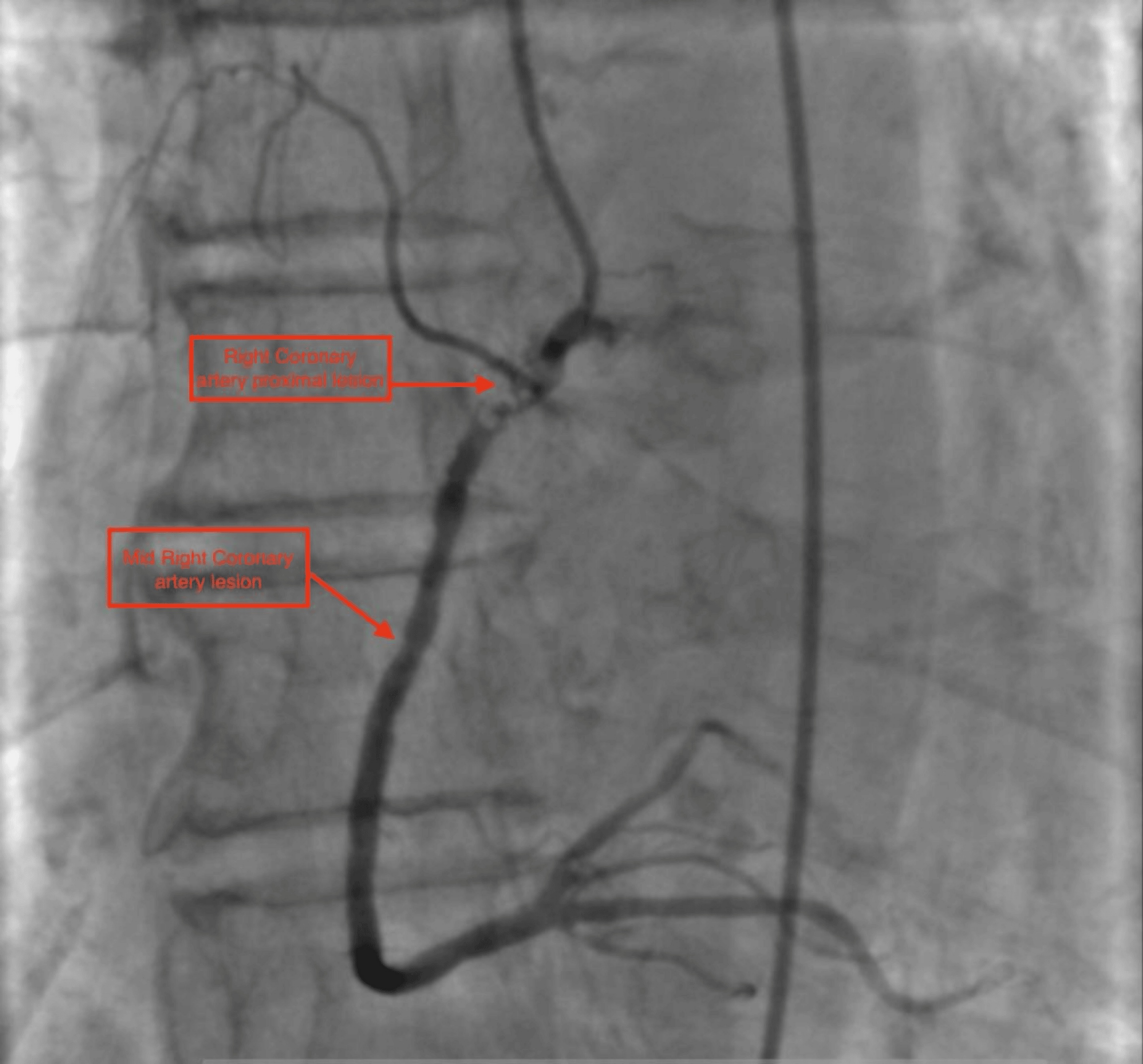
Right circulation shows a 99% stenotic lesion involving the ostia and proximal part of the RCA and another 80% stenotic lesion in the mid-RCA. RCA, right coronary artery

Angiography of the right-sided coronary circulation revealed a severe, highly calcified stenotic lesion at the ostia and proximal part of the RCA, estimated to be 99% stenosed, along with an additional 80% stenotic lesion in the mid RCA (Figure 3).

An intravascular shockwave lithotripsy was performed, followed by intravascular ultrasound-guided angioplasty using standard balloon dilatation via a non-compliant balloon, followed by deployment of a drug-eluting Onyx stent measuring 3.5 x 26 mm in the ostial and proximal part of the RCA and another 3.5 x 18 mm drug-eluting stent in the mid-RCA lesion. After stent deployment, assessment via the Thrombolysis in Myocardial Infarction (TIMI) Flow Grading System revealed a grade 3 TIMI and 0% residual stenosis observed in both lesions. Unfortunately, soon after angioplasty, the patient developed symptomatic bradycardia, and follow-up ECG revealed a junctional rhythm with a heart rate of 58 bpm. A temporary pacemaker was placed, and the patient was transferred to the cardiac ICU for further monitoring and management.

Outcome

**Figure 4:**
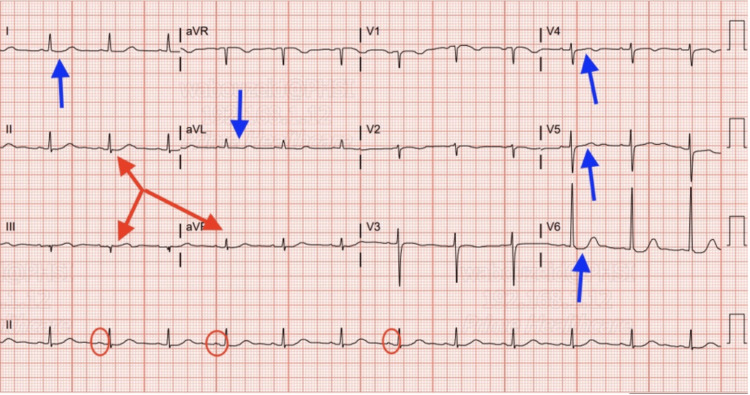
ECG shows sinus rhythm (red circles) with a heart rate of 75 beats per minute and reversing ST and T wave changes (red and blue arrows) that were previously shown in the initial ECG.

The patient remained hemodynamically stable in the cardiac ICU, and dual antiplatelet therapy (DAPT) consisting of aspirin and ticagrelor and high-intensity statins were resumed. Beta-blocker therapy was initially deferred due to the presence of the junctional rhythm. The following day, a follow-up ECG revealed a sinus rhythm with a rate of 75 bpm, along with reversed ST and T wave changes that were present in the initial ECG (Figure 4).

The transvenous pacemaker was later removed. The patient remained hemodynamically stable, denied any recurrent chest pain, and had no more junctional beats noted throughout her hospital stay. A transthoracic echocardiogram (ECHO) shows a normal ejection fraction of 60%, with normal right and left ventricular systolic and diastolic function, and mild aortic and tricuspid regurgitation. Later on, the patient was discharged home on DAPT, high-intensity statin, amlodipine, and lisinopril for the treatment of hypertension.

Latest follow-up

The temporary pacemaker was removed, and the patient maintained sinus rhythm during the rest of her hospital stay. An ECHO was performed, which revealed inferior wall hypokinesis with a left ventricular ejection fraction of 60-65%. Unfortunately, there was no prior ECHO for comparison. During her follow-up visit in the outpatient clinic after one week, the patient denied any new symptoms and was compliant with her medications. A repeat ECHO was performed at a six-month interval, which showed no regional wall abnormalities and a normal ejection fraction.

## Discussion

Radiation treatment-related cardiac toxicity has been studied in thoracic malignancies such as lung, breast, and gastric cancers, and its clinical manifestations depend on patient-related factors and radiation exposure [3]. Adjuvant radiation therapy following breast cancer surgery improves locoregional control and survival; however, inadvertent cardiac exposure increases the risk of radiation-induced cardiac injury [3]. Survivors of breast cancer are therefore at an increased risk of long-term radiation-related cardiotoxicity, significantly affecting quality of life [3]. One study demonstrated that radiation exposure is associated with increased coronary calcium scores and development of both calcified and non-calcified plaques [5]. Because vascular calcification progresses silently, most patients remain asymptomatic for years [5]. Lesions commonly involve the proximal LAD artery and RCA [5]. In our case, the patient developed STEMI 30 years after radiation therapy, likely due to rupture of a previously calcified atherosclerotic plaque in the RCA.

CAC is highly age- and sex-dependent, with prevalence increasing significantly in older populations [6]. Multiple cardiovascular risk factors contribute to CAC development, including dyslipidemia, hypertension, diabetes mellitus, chronic kidney disease, systemic inflammation, and prior radiation exposure [6].

Radiation therapy is associated with increased long-term cardiovascular mortality, with coronary artery disease being the most common manifestation [2,7]. The risk of CAD increases in a dose-dependent manner with mean heart radiation exposure, and patients with left-sided breast cancer have higher cardiac exposure compared with right-sided disease, leading to increased cardiovascular events [2].

Kirresh et al. demonstrated that radiation-induced coronary artery disease represents a complex clinical entity with long latency and diagnostic challenges, reinforcing the long-term vascular effects of thoracic radiotherapy [8].

A study by Hancock et al. demonstrated that radiation-associated MI risk is highest in younger patients and decreases with age at exposure, highlighting the long latency of radiation-induced coronary disease [9]. This raises important considerations regarding timing of cardiovascular surveillance following mediastinal irradiation [9].

Clarke et al. similarly demonstrated increased ischemic heart disease in patients receiving cardiac radiation exposure [7]. The underlying mechanisms include both macrovascular atherosclerotic changes and microvascular injury [7]. Additionally, Darby et al. demonstrated a linear increase in major coronary events with increasing mean heart dose, with risk detectable within five years of exposure [2].

Cuzick et al. showed increased cause-specific mortality in long-term breast cancer survivors treated with radiotherapy, supporting late cardiovascular complications as a major determinant of long-term outcomes [10].

Vascular calcification is a regulated, cell-mediated process involving osteogenic transformation of vascular smooth muscle cells, apoptosis, and imbalance of calcification inhibitors such as fetuin-A and pyrophosphate [11]. CAC is strongly associated with adverse cardiovascular outcomes and mortality, and its progression correlates with an increased risk of major events [6].

CT coronary angiography and coronary artery calcium scoring are widely used non-invasive methods for assessing coronary atherosclerotic burden, with CAC serving as a strong predictor of cardiovascular risk [6].

Percutaneous coronary intervention (PCI) becomes more challenging in the presence of coronary artery calcification (CAC). A meta-analysis of interventional cardiology studies demonstrated that calcified coronary lesions are associated with worse procedural and long-term outcomes following PCI [12]. Patients with severe coronary calcification had higher rates of adverse cardiovascular events, including mortality, MI, and repeat revascularization, as well as lower rates of complete revascularization compared with patients without significant calcification [12]. As a result, CAC is considered a marker of more complex coronary anatomy and poorer clinical outcomes following PCI [12].

Table 1 summarizes some reports and reviews for a similar condition [8,13-15].

**Table 1:** Previous cases and reviews on radiation-induced coronary artery disease.

Author, Year	Underlying Malignancy	Radiation Site / Dose	Latency After Radiation	Coronary Findings / Calcification	Clinical Presentation	Management	Outcome
McEniery et al., 1987 [13]	Hodgkin lymphoma and other mediastinal malignancies	Mediastinal radiation	Approximately 15 years	Severe proximal coronary artery disease with calcific changes, including RCA involvement	Angina and myocardial infarction	CABG or PCI	Symptomatic improvement after revascularization
Om et al., 1992 [14]	Hodgkin lymphoma	Chest/mediastinal radiation	Approximately 10–20 years	Proximal coronary stenosis with severe calcification	Symptomatic coronary artery disease	Surgical or percutaneous revascularization	Clinical improvement reported
Ruiz et al., 2018 [15]	Hodgkin lymphoma	Mediastinal radiation	16 years	Multivessel heavily calcified coronary artery disease	Acute coronary syndrome	PCI	Successful intervention with recovery
Kirresh et al., 2022 [8]	Prior thoracic malignancy	Mediastinal radiotherapy	Several decades	Diffuse heavily calcified coronary artery disease	Exertional angina and ischemia	Complex PCI considered; CABG discussed	Managed with revascularization strategy

## Conclusions

Our case, together with similar reported cases, highlights the potential serious cardiovascular consequences of thoracic radiation therapy. Radiation-induced heart disease is well established in the literature, with coronary artery calcification representing a major manifestation associated with significant morbidity, mortality, and procedural challenges during PCI. Therefore, greater emphasis should be placed on research aimed at preventing radiation-induced coronary artery disease and establishing strategies for early detection before severe complications occur. Potential approaches include using the lowest effective radiation dose with adequate cardiac protection during therapy, as well as implementing coronary CT angiography with calcium scoring as a screening tool. Additionally, investigating both conventional coronary artery disease therapies and novel anti-calcification treatments in asymptomatic high-risk patients may help prevent or slow disease progression.

## References

[1] Ibanez B, James S, Agewall S (2018). 2017 ESC Guidelines for the management of acute myocardial infarction in patients presenting with ST-segment elevation: The Task Force for the management of acute myocardial infarction in patients presenting with ST-segment elevation of the European Society of Cardiology (ESC). Eur Heart J.

[2] Darby SC, Ewertz M, McGale P (2013). Risk of ischemic heart disease in women after radiotherapy for breast cancer. N Engl J Med.

[3] Lenneman CG, Sawyer DB (2016). Cardio-oncology: an update on cardiotoxicity of cancer-related treatment. Circ Res.

[4] Armenian SH, Lacchetti C, Barac A (2017). Prevention and monitoring of cardiac dysfunction in survivors of adult cancers: ASCO clinical practice guideline. J Clin Oncol.

[5] Rademaker J, Schöder H, Ariaratnam NS, Strauss HW, Yahalom J, Steingart R, Oeffinger KC (2008). Coronary artery disease after radiation therapy for Hodgkin's lymphoma: coronary CT angiography findings and calcium scores in nine asymptomatic patients. AJR Am J Roentgenol.

[6] Budoff MJ, Hokanson JE, Nasir K (2010). Progression of coronary artery calcium predicts all-cause mortality. JACC Cardiovasc Imaging.

[7] Clarke M, Collins R, Darby S (2005). Effects of radiotherapy and extent of surgery for early breast cancer on local recurrence and 15-year survival: overview of randomized trials. Lancet.

[8] Kirresh A, White L, Mitchell A (2022). Radiation-induced coronary artery disease: a difficult clinical conundrum. Clin Med (Lond).

[9] Hancock SL, Tucker MA, Hoppe RT (1993). Factors affecting late mortality from heart disease after treatment of Hodgkin's disease. JAMA.

[10] Cuzick J, Stewart H, Rutqvist L (1994). Cause-specific mortality in long-term survivors of breast cancer who participated in trials of radiotherapy. J Clin Oncol.

[11] Demer LL, Tintut Y (2008). Vascular calcification: pathobiology of a multifaceted disease. Circulation.

[12] Généreux P, Madhavan MV, Mintz GS (2014). Ischemic outcomes after coronary intervention of calcified vessels in acute coronary syndromes. Pooled analysis from the HORIZONS-AMI (Harmonizing Outcomes With Revascularization and Stents in Acute Myocardial Infarction) and ACUITY (Acute Catheterization and Urgent Intervention Triage Strategy) TRIALS. J Am Coll Cardiol.

[13] McEniery PT, Dorosti K, Schiavone WA, Pedrick TJ, Sheldon WC (1987). Clinical and angiographic features of coronary artery disease after chest irradiation. Am J Cardiol.

[14] Om A, Ellahham S, Vetrovec GW (1992). Radiation-induced coronary artery disease. Am Heart J.

[15] Ruiz CR, Mesa-Pabón M, Soto K, Román JH, López-Candales A (2018). Radiation-induced coronary artery disease in young patients. Heart Views.

